# Genome-scale data suggest reclassifications in the *Leisingera-Phaeobacter* cluster including proposals for *Sedimentitalea* gen. nov. and *Pseudophaeobacter* gen. nov.

**DOI:** 10.3389/fmicb.2014.00416

**Published:** 2014-08-11

**Authors:** Sven Breider, Carmen Scheuner, Peter Schumann, Anne Fiebig, Jörn Petersen, Silke Pradella, Hans-Peter Klenk, Thorsten Brinkhoff, Markus Göker

**Affiliations:** ^1^Department of Biology of Geological Processes - Aquatic Microbial Ecology, Institute for Chemistry and Biology of the Marine Environment (ICBM), University of OldenburgOldenburg, Germany; ^2^Department of Microorganisms, Leibniz Institute DSMZ - German Collection of Microorganisms and Cell CulturesBraunschweig, Germany

**Keywords:** marine microbiology, *Roseobacter* group, phylogenomics, supermatrix, gene content, genus boundaries

## Abstract

Earlier phylogenetic analyses of the marine *Rhodobacteraceae* (class *Alphaproteobacteria*) genera *Leisingera* and *Phaeobacter* indicated that neither genus might be monophyletic. We here used phylogenetic reconstruction from genome-scale data, MALDI-TOF mass-spectrometry analysis and a re-assessment of the phenotypic data from the literature to settle this matter, aiming at a reclassification of the two genera. Neither *Phaeobacter* nor *Leisingera* formed a clade in any of the phylogenetic analyses conducted. Rather, smaller monophyletic assemblages emerged, which were phenotypically more homogeneous, too. We thus propose the reclassification of *Leisingera nanhaiensis* as the type species of a new genus as *Sedimentitalea nanhaiensis* gen. nov., comb. nov., the reclassification of *Phaeobacter arcticus* and *Phaeobacter leonis* as *Pseudophaeobacter arcticus* gen. nov., comb. nov. and *Pseudophaeobacter leonis* comb. nov., and the reclassification of *Phaeobacter aquaemixtae, Phaeobacter caeruleus*, and *Phaeobacter daeponensis* as *Leisingera aquaemixtae* comb. nov., *Leisingera caerulea* comb. nov., and *Leisingera daeponensis* comb. nov. The genera *Phaeobacter* and *Leisingera* are accordingly emended.

## Introduction

Bacteria belonging to the *Roseobacter* group are presumed to form a monophyletic group within the *Rhodobacteraceae* (*Alphaproteobacteria*), the great majority of them being of marine origin, which is reflected by their absolute requirement of sodium ions (Brinkhoff et al., 2008). Members of this group constitute up to 25% of the total bacterial community in a large variety of habitats (Brinkhoff et al., 2008). Because of their high abundance and their adaptive life style, they are thought to play a major role in global chemical cycles. Some of the important traits of the *Roseobacter* group are the use of a multitude of organic compounds, sulfur oxidation, oxidation of carbon monoxide, DMSP demethylation, the production of secondary metabolites (Buchan et al., 2005; Moran et al., 2007; Brinkhoff et al., 2008) and their affiliation with the cohort of aerobic anoxygenic phototrophic bacteria (Yurkov and Beatty, 1998).

Many representatives of the *Roseobacter* group can be cultivated in the lab, which is one of the reasons why the group is continuously growing. Until 2008, 38 genera were described (Brinkhoff et al., 2008), and at the time of writing the group contained at least 54 genera and 135 species with validly published names. The huge amount of genera and species reflects the physiological and genetic diversity within this group (Buchan et al., 2005) and makes it necessary to monitor the classification of previously published species and genera. Several reclassifications were already necessary within the *Roseobacter* group, e. g., for the genera *Ruegeria* (Uchino et al., 1998; Arahal et al., 2005; Martens et al., 2006) and *Roseobacter* (Martens et al., 2006).

The genus *Leisingera* was proposed by Schaefer et al. (2002) and currently consists of three species, *Leisingera methylohalidivorans* (Schaefer et al., 2002), *Leisingera aquimarina* (Vandecandelaere et al., 2008), and *Leisingera nanhaiensis* (Sun et al., 2010). The strains belonging to these species were isolated from sea water, a marine electro-active biofilm and marine sandy sediment, respectively. Phylogenetic trees for the trimethylamine monooxygenase (*tmm*) and the gamma-glutamyl-methylamine synthetase (Chen, 2012) showed that *L. nanhaiensis* is more distantly related to the other *Leisingera* species. This was confirmed by recent 16S rRNA gene sequence analyses (even though largely unresolved) and preliminary genomic analyses using the Genome-to-Genome Distance Calculator (GGDC; Auch et al., 2010a,b; Meier-Kolthoff et al., 2013) of diverse *Phaeobacter* and *Leisingera* type strains (Beyersmann et al., 2013; Buddruhs et al., 2013a; Dogs et al., 2013a,b; Freese et al., 2013; Riedel et al., 2013; Breider et al., 2014), suggesting the need for reclassification of *L. nanhaiensis*. Digital DNA:DNA hybridization (DDH) estimates as delivered by the GGDC were preferred over Average Nucleotide Identity (ANI) estimates because they provide higher correlations with traditional DDH results than do any of the ANI implementations (Auch et al., 2010b; Meier-Kolthoff et al., 2013).

The genus *Phaeobacter* was introduced by Martens et al. (2006) and currently comprises the species *Phaeobacter aquaemixtae* (Park et al., 2014), *Phaeobacter arcticus* (Zhang et al., 2008), *Phaeobacter caeruleus* (Vandecandelaere et al., 2009), *Phaeobacter daeponensis* (Yoon et al., 2007), *Phaeobacter inhibens* (Martens et al., 2006), *Phaeobacter leonis* (Gaboyer et al., 2013), and *Phaeobacter gallaeciensis* (Ruiz-Ponte et al., 1998; Martens et al., 2006), which is the type species, its type strain being BS107^T^ = CIP 105210^T^ = DSM 26640^T^ but not DSM 17395 (Buddruhs et al., 2013b). The *Phaeobacter* species were isolated from a mixing zone of the ocean and a freshwater spring, marine arctic sediment, a marine electro-active biofilm, tidal flat sediment, a tidal mud flat, marine surface sediment and rearings and collectors of the scallop *Pecten maximus*, respectively. Recently, *Phaeobacter* strains retrieved a lot of interest because of their production of various secondary metabolites (e.g., Berger et al., 2011). Analyses of the 16S rRNA gene and in some publications also preliminary genomic analyses were in conflict with the current classification (Jin et al., 2011; Beyersmann et al., 2013; Buddruhs et al., 2013a; Dogs et al., 2013a,b; Freese et al., 2013; Gaboyer et al., 2013; Riedel et al., 2013; Breider et al., 2014; Liu et al., 2014; Park et al., 2014). These analyses mostly showed that *P. aquaemixtae, P. caeruleus, P. daeponensis, Leisingera methylohalodivorans*, and *L. aquimarina* form a clade, *P. arcticus* and *P. leonis* comprise a distinct monophyletic group, and *P. gallaeciensis* and *P. inhibens* form a third clade.

Thus, the two genera *Leisingera* and *Phaeobacter* appear intermixed. When 16S rRNA genes are insufficiently resolved, it is necessary to conduct phylogenetic analyses with additional genes. In many respects, using genome-scale data is the most promising approach (Klenk and Göker, 2010). As shown, e.g., in a series of studies using the DSMZ phylogenomics pipeline (Spring et al., 2010; Anderson et al., 2011; Abt et al., 2012, 2013; Frank et al., 2014; Stackebrandt et al., 2014; Verbarg et al., 2014), the more characters are assembled, the better statistically supported are the resulting phylogenies. Thus, phylogenomics has the potential to yield a more stable taxonomy, given the general goal that the taxonomic classification should summarize the phylogeny of the organisms (Klenk and Göker, 2010; Wiley and Lieberman, 2011; Göker and Klenk, 2013).

According to the aforementioned preliminary genomic analyses, we here re-investigated the phylogenetic relationships of *Phaeobacter* and *Leisingera* spp. using a variety of methods applied to genome-scale data, for determining monophyletic groups that are stable under a broad range of conditions. We also analyzed the relevant type strains using Matrix-assisted laser desorption/ionization time-of-flight mass spectronomy (MALDI-TOF MS) technology and re-assessed the published phenotypic information for providing descriptions of new or redefined taxa, including a recalculation of the G+C content from the genome data (Meier-Kolthoff et al., 2014).

## Materials and methods

Comprehensive samples of 16S rRNA gene data available from the Living Tree Project (LTP; Munoz et al., 2011), version s111, were used to determine the range of other genera that should be compared with the genera *Phaeobacter* and *Leisingera*. Because as yet the LTP phylogeny does not contain branch-support values, it has only limited use for directly assessing evolutionary relationships. We thus extracted the *Rhodobacteraceae* part of the LTP alignment, deleted all resulting gap-only alignment columns and phylogenetically analyzed the resulting matrix including bootstrapping as described below. Taxon sampling for all further, more detailed analyses was based on this initial assessment.

Protein sequences from the 14 available type-strain genomes of *Leisingera, Phaeobacter, Ruegeria* and outgroup (*Oceanibulbus, Roseobacter*, and *Sediminimonas*) species (Beyersmann et al., 2013; Buddruhs et al., 2013a; Dogs et al., 2013a,b; Freese et al., 2013; Riedel et al., 2013; Breider et al., 2014) were retrieved from the IMG website (http://img.jgi.doe.gov/cgi-bin/w/main.cgi) (*L. aquimarina* DSM 24565^T^, ID 2516653083 = AXBE00000000; *L. methylohalidivorans* MB2^T^, ID 2512564009 = CP006773/CP006774/CP006775; *L. nanhaiensis* NH52F^T^, ID 2512047090 = AXBG00000000; *P. arcticus* DSM 23566^T^; ID 2516653081 = AXBF00000000; *P. caeruleus* 13^T^, ID 2512047087 = AXBI00000000; *P. daeponensis* TF-218^T^, ID 2516493020 = AXBD00000000; *P. inhibens* T5^T^, ID 2516653078 = AXBB00000000; *Sediminimonas qiaohouensis* DSM 21189^T^, ID 2523533612 = AUIJ00000000) or from NCBI (*Oceanibulbus indolifex* HEL-45^T^, ABID00000000; *P. gallaeciensis* CIP105210^T^, AOQA01000000; *Roseobacter denitrificans* Och 114^T^, CP000362, CP000464, CP000465, CP000466, CP000467; *Roseobacter litoralis* Och 149^T^, CP002623, CP002624, CP002625, CP002626; *Ruegeria lacuscaerulensis* ITI-1157^T^, ACNX00000000; ACNX00000000; *Ruegeria pomeroyi* DSS-3^T^, CP000031, CP000032).

The genome sequences were phylogenetically investigated using the DSMZ phylogenomics pipeline as previously described (Spring et al., 2010; Anderson et al., 2011; Göker et al., 2011; Abt et al., 2012, 2013; Frank et al., 2014; Stackebrandt et al., 2014; Verbarg et al., 2014). In brief, clusters of orthologs were determined with a re-implementation of the OrthoMCL algorithm (Li et al., 2003) using NCBI BLAST version 2.2.25 (Altschul et al., 1997) and in conjunction with MCL version 11-294 (http://micans.org/mcl/) under default settings. OrthoMCL clusters containing inparalogs were reduced by selecting the most “central” sequence from each genome (the one with highest sum of BLAST scores), aligned using MUSCLE version 3.8.31 (Edgar, 2004), and the alignments filtered with the program scan_orphanerrs from the RASCAL package version 1.3.4 (Thompson et al., 2003) to remove orphan sequences as well as GBLOCKS version 0.91b (Castresana, 2000) to remove poorly aligned columns. Here, three distinct supermatrices (concatenated alignments) were generated: (i) using the “core genes” only, i.e., those alignments containing sequences from all genomes, (ii) a “full” matrix using all alignments comprising at least four sequences, (iii) the same matrix but filtered with MARE (http://mare.zfmk.de) (Meusemann et al., 2010) without removing organisms. Additionally, two smaller matrices of preselected genes were analyzed, using the distinct sets of 31 genes, respectively, suggested by Ciccarelli et al. (2006) and Wu and Eisen (2008). The OrthoMCL clusters were also converted to an ortholog-content matrix representing the presence or absence of a gene within a certain genome and clusters of orthologs. Further, clusters of homologous sequences were determined using a re-implementation of the TribeMCL algorithm (Enright et al., 2002), applying an *e*-value threshold of 10^−5^ and an MCL inflation parameter of 2.0. The clusters of homologs were converted to a gene-content matrix in analogy to the ortholog-content matrix.

As no genomic data were available for the other organisms of interest (further *Ruegeria* species, *Litorimicrobium taeanense* (Jin et al., 2011), *Phaeobacter aquamixtae* (Park et al., 2014), *P. leonis* (Gaboyer et al., 2013), *Puniceibacterium antarcticum* (Liu et al., 2014), and *Seohaeicola saemankumensis* (Yoon et al., 2009), their position was assessed by 16S rRNA gene sequences only. These were analyzed unconstrained as well as constrained by enforcing the monophyly of the maximally supported groups from the supermatrix analysis. As the 16S rRNA gene analysis contained more species than the phylogenomic analyses, the supermatrix tree yields a backbone constraint, which enforces only the relative positioning of the species contained in all data matrices. The 16S rRNA gene alignment used was again the one from the LTP version s111, from which taxa not of interest were removed (with deletion of all resulting gap-only alignment columns) and to which the sequences of *P. aquamixtae* (KF554505), *P. leoni*s (HE661585), and *P. antarcticum* (JX070673) were aligned using POA version 2.0 (Lee et al., 2002).

Maximum likelihood (ML) (Felsenstein, 1981) and maximum-parsimony (MP) (Fitch, 1977; Goloboff, 2003) phylogenetic trees were inferred from the data matrices as previously described (Spring et al., 2010; Anderson et al., 2011; Göker et al., 2011; Abt et al., 2012, 2013; Frank et al., 2014; Stackebrandt et al., 2014; Verbarg et al., 2014). The Pthreads-parallelized RAxML package version 7.2.8 (Stamatakis, 2006) was used for ML, applying fast bootstrapping in conjunction with the autoMRE bootstopping criterion (Pattengale et al., 2010) and subsequent search for the best tree (Stamatakis et al., 2008). Tree searches under the MP criterion were conducted with PAUP* version 4b10 (Swofford, 2002) using 100 rounds of random sequence addition and subsequent TBR branch swapping, saving no more than 10 best trees per round and collapsing potential zero-length branches during tree search. MP bootstrap support was calculated with PAUP^*^ using 1000 replicates with 10 rounds of heuristic search per replicate. For each supermatrix, the best ML amino-acid substitution model was determined beforehand by comparing the resulting log likelihoods on a MP starting tree. For the ortholog-content and the gene-content matrices, the BINGAMMA model as implemented in RAxML was used, and for the rRNA gene matrices the GTRGAMMA model. The phylogenomic trees were checked for long-branch attraction artifacts (Felsenstein, 2004; Bergsten, 2006) using selected long-branch extraction (Siddall and Whiting, 1999) experiments. To assess the significance of phylogenetic conflict, if any, between data matrices, paired-site tests as implemented in RAxML and PAUP^*^ were conducted, comparing the best tree(s) from unconstrained search with the best ones from a search (backbone-) constrained for the well-supported parts of the topology obtained via one to several other analyses.

The G+C content of all species was determined from the genome sequences, allowing for higher precision than the wet-lab methods. The values were taken from previous studies (Beyersmann et al., 2013; Buddruhs et al., 2013a; Dogs et al., 2013a,b; Freese et al., 2013; Riedel et al., 2013; Breider et al., 2014; Frank et al., 2014).

Whole-cell protein extracts of the type strains of *Phaeobacter* and *Leisingera* as well as those of the neighboring genera *Litorimicrobium, Nautella, Oceanibulbus, Sediminimonas, Salinihabitans, Seohaeicola, Roseobacter*, and *Ruegeria* were analyzed by MALDI-TOF MS (Maier and Kostrzewa, 2007) using a Microflex L20 mass spectrometer (Bruker Daltonics) equipped with a N_2_ laser. Sample preparation for MALDI-TOF MS protein analysis was carried out according to the ethanol/formic acid extraction protocol recommended by Bruker Daltonics as described in detail by Tóth et al. (2008). The MALDI-TOF mass spectra were analyzed with the BioTyper software (version 3.1, Bruker Daltonics).

## Results

The comprehensive 16S rRNA gene alignment for the *Rhodobacteraceae* contained 245 organisms and 1314 characters. The resulting ML and MP trees had a log likelihood of -32912.16 and a number of steps of 6329, respectively, and are shown in Supplementary File 1 together with the bootstrapping values. As expected, the 16S rRNA gene analysis overall suffered from poor resolution (average branch support 45.3% under ML, 39.7% under MP). The *Leisingera* and *Phaeobacter* species were distributed over several clusters, hence none of the two genera formed a monophyletic group. With the exception of the strongly supported (99/98%) sister-group relationship between *P. arcticu*s and *P. leonis*, the moderately supported (86/92%) group comprising *L. methylohalidivorans* and *L. aquimarina* and the weakly supported (69/<60%) group containing *P. aquaemixtae, P. caeruleus*, and *P. daeponensis*, the internal edges of these clades were unsupported. Other species that might potentially form the sister group of any of the *Phaeobacter* and *Leisingera* clades included *Pelagicola litoralis* and *P. antarcticum*. In contrast, *Nautella italica, S. saemankumensis*, and *L. taeanense* were placed in more isolated positions, whereas *S. qiaohouensis* and *Salinihabitans flavidus* formed a moderately supported (89/62%) clade that comprised the sister group of the cluster containing *Ruegeria*. So the evidence that any of these genera were intermixed with either *Leisingera* or *Phaeobacter* was negligible, but to assume a close relationship of other genera with *Leisingera* or *Phaeobacter* would be even more speculative. For this reason, the forthcoming analyses were restricted to the genera *Leisingera, Pelagicola, Phaeobacter*, and *Puniceibacterium*. Additionally, only *Litorimicrobium, Nautella, Ruegeria, Salinihabitans, Sediminimonas*, and *Seohaeicola* were included to serve as a close outgroup and *Roseobacter* and *Oceanibulbus* for rooting the tree, yielding 14 organisms in the phylogenomic and 28 organisms in an additional 16S rRNA analysis.

**Figure 1:**
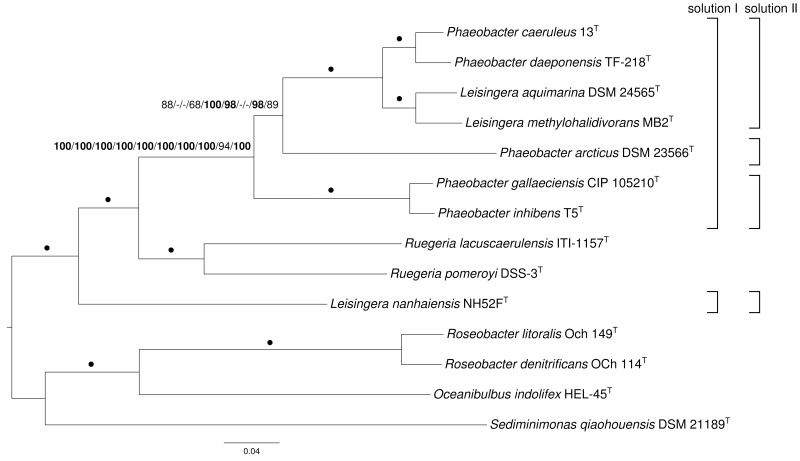
**Phylogenetic tree inferred from the core-gene matrix under the maximum likelihood (ML) criterion and rooted with *Oceanibulbus, Roseobacter* and *Sediminimonas***. The branches are scaled in terms of the expected number of substitutions per site. Numbers above the branches (from left to right) are bootstrapping support values (if larger than 60%) from (i) ML core-genes; (ii) maximum-parsimony (MP) core-genes; (iii) ML unfiltered supermatrix; (iv) MP unfiltered supermatrix; (v) ML MARE-filtered supermatrix; (vi) MP MARE-filtered supermatrix; (vii) ML “Ciccarelli” matrix; (viii) MP “Ciccarelli” matrix; (ix) ML “Wu-Eisen” matrix; (x) MP “Wu-Eisen” matrix analysis. Values larger than 95% are shown in bold; dots indicate branches with maximum support under all settings. On the right side two potential new taxonomic arrangements into genera are shown that are in agreement with the tree.

The core-gene amino-acid supermatrix comprised 1550 genes and 502,216 characters, whereas the “full” supermatrix contained 4582 genes and 1,390,199 characters before, 2527 genes and 767,593 characters after cleaning with MARE. The “Ciccarelli” data matrix contained 7811 characters, whereas the “Wu-Eisen” matrix comprised 7905 characters. For all five matrices, the selected model was PROTGAMMALGF [the LG model of amino acid evolution (Le and Gascuel, 2008) in conjunction with gamma-distributed substitution rates (Yang, 1993) and empirical amino acid frequencies]. The resulting trees had log likelihoods of (i) -5132414.78, (ii) -12814043.60, (iii) -7798812.71, (iv) -57494.18 and (v) -53469.14, respectively. The core-gene, MARE-filtered supermatrix as well as “Wu-Eisen” trees were topologically identical. This topology is shown in Figure 1 together with ML and MP bootstrap support values from all five supermatrix analyses if larger than 60%. The tree inferred from the unfiltered supermatrix and “Ciccarelli” matrix showed a distinct grouping of *P. arcticus* DSM 23566^T^, i.e., as sister group of the clade comprising *P. inhibens* T5^T^ and *P. gallaeciensis* CIP 105210^T^. The best MP trees found had lengths of (i) 714,607, (ii) 1,749,584, (iii) 1,091,094, (iv) 6225, (v) 5499 steps, respectively, (not counting counting uninformative characters) and were topologically identical to the ML core-gene and MARE-filtered supermatrix trees. The “Ciccarelli” tree showed yet another grouping of *P. arcticus* DSM 23566^T^, i.e., as sister group of the clade comprising *L. aquimarina* DSM 24565^T^, *L. methylohalidivorans* MB2^T^, *P. caeruleus* 13^T^, *P. daeponensis* TF-218^T^, *P. inhibens* T5^T^, and *P. gallaeciensis* CIP 105210^T^. Support was maximum (100%) for all branches under ML and MP but the previously described deviating ones (Figure 1). The trees agreed regarding a maximally supported monophyletic group comprising *P. daeponensis, P. caeruleus, L. methylohalidivorans* as well as *L. aquimarina*, regarding another clade with maximum support containing *P. gallaeciensis* and *P. inhibens*, and regarding the placement of *Ruegeria* spp. as sister group of *Leisingera* and *Phaeobacter* to the exclusion of *L. nanhaiensis*. Removal of the outgroup and subsequent phylogenetic inference yielded trees with the same topology that would have been obtained by pruning the outgroup from the tree depicted in Figure 1 (data not shown), indicating that the position of *L. nanhaiensis* is not due to long branch attraction.

**Figure 2:**
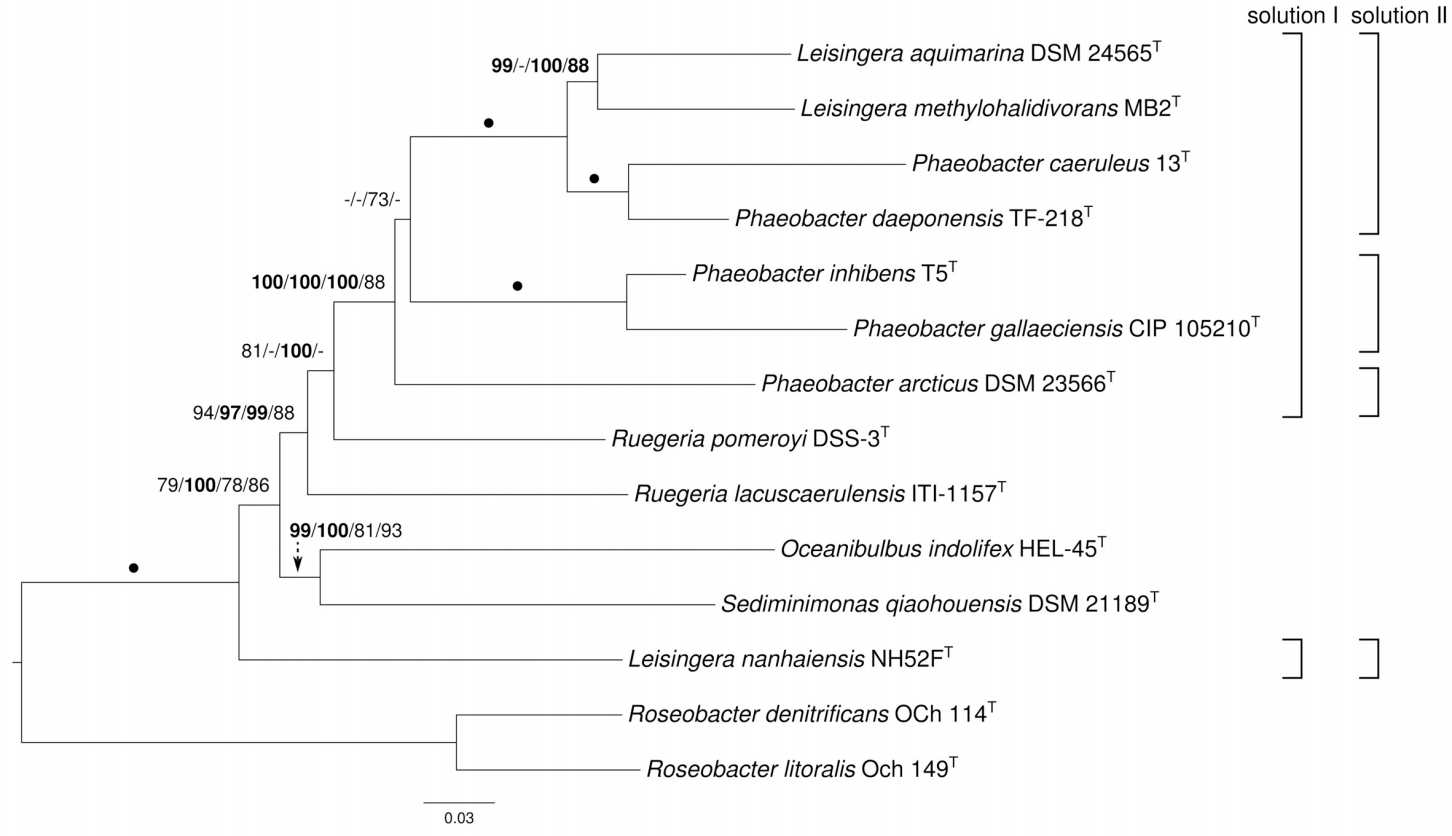
**Phylogeny inferred from the ortholog-content matrix under the maximum likelihood (ML) criterion and rooted with *Roseobacter***. The branches are scaled in terms of the expected number of substitutions per site. Numbers above the branches (from left to right) are bootstrapping support values (if larger than 60%) from (i) ML ortholog-content matrix; (ii) maximum-parsimony (MP) ortholog-content matrix; (iii) ML gene-content matrix; (iv) MP gene-content matrix analysis. Values larger than 95% are shown in bold; dots indicate branches with maximum support under all settings. On the right side two potential new taxonomic arrangements into genera are shown that are in agreement with the tree.

The ortholog-content matrix contained 13,676 characters, and the resulting best trees had a log likelihood of -77923.13 and a length of 19,909 steps, respectively. The gene-content matrix comprised 9844 characters and yielded best trees with a log likelihood of -54954.97 and a parsimony score of 13,580, respectively. Both ML trees were topologically identical and are shown in Figure 2 with bootstrap support values for ML and MP if larger than 60%. The MP gene-content tree showed the monophyly of *Ruegeria*, whereas *P. arcticus* DSM 23566^T^ was grouped as in the supermatrix tree (see Figure 1). Like the supermatrix trees, a maximally supported clade comprising *P. daeponensis, P. caeruleus, L. methylohalidivorans* as well as *L. aquimarina* and another one containing *P. gallaeciensis* and *P. inhibens* were revealed. In addition to *Ruegeria* spp., in the gene- and ortholog-content trees *Oceanibulbus* and *Sediminimonas* were indicated as more closely related than *L. nanhaiensis* to the remaining *Leisingera* and *Phaeobacter* species. None of the branches in conflict with the supermatrix trees were particularly well supported.

**Figure 3:**
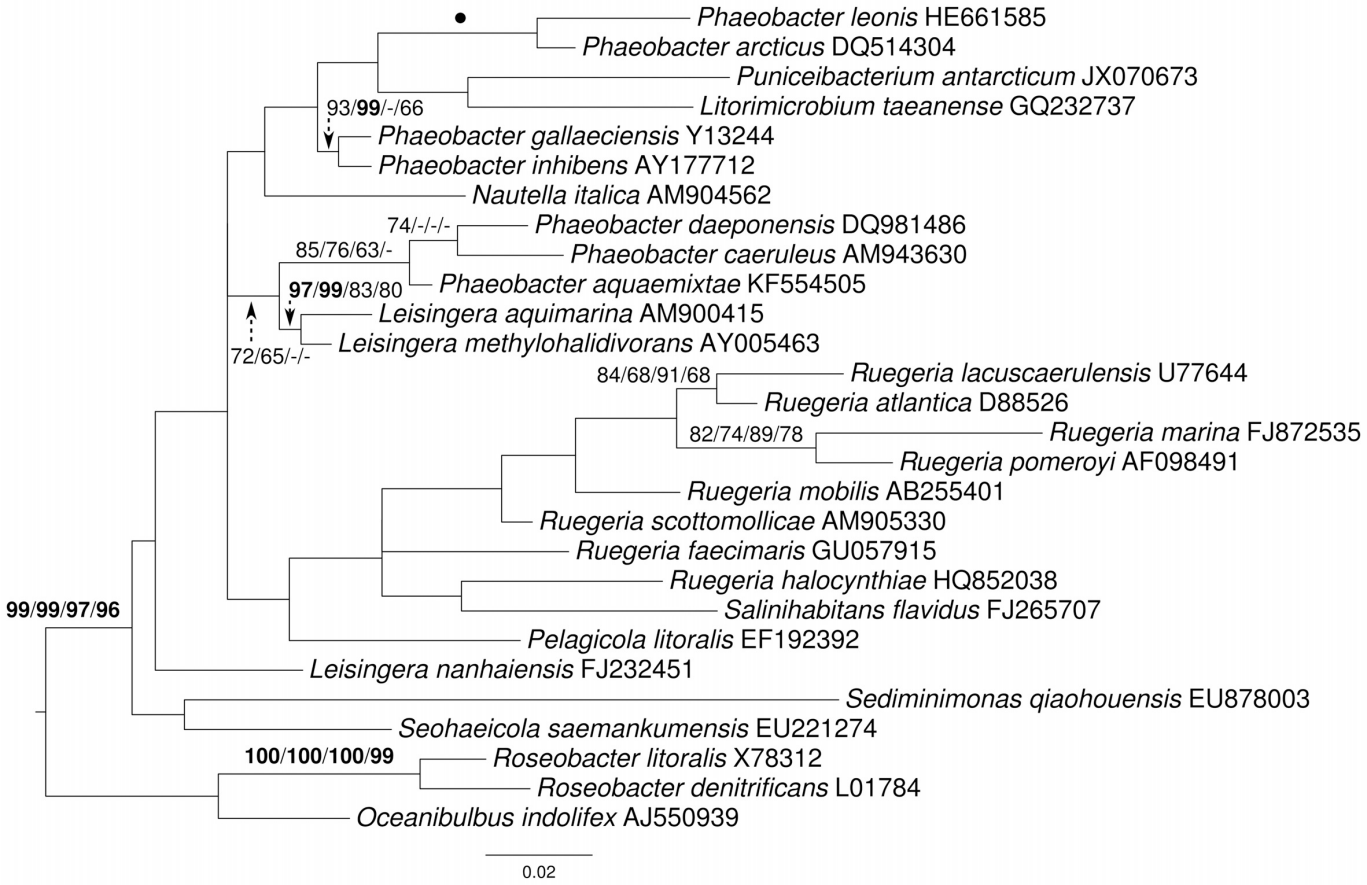
**Phylogeny inferred from the 16S rRNA gene matrix under the maximum likelihood (ML) criterion and the maximally supported branches of the topology depicted in Figure 1 as backbone constraint**. Rooting was done with *Oceanibulbus* and *Roseobacter*. The branches are scaled in terms of the expected number of substitutions per site. Numbers above the branches (from left to right) are bootstrapping support values (if larger than 60%) from (i) constrained ML, (ii) constrained maximum-parsimony (MP), (iii) unconstrained ML, and (iv) unconstrained MP analysis. Values larger than 95% are shown in bold; dots indicate branches with maximum support under all settings.

The rRNA gene matrix of selected organisms contained 1503 characters and yielded a highest likelihood of -6252.76 and a minimal length of 790 steps in unconstrained search, -6292.42 and 807 steps in constrained search. The constrained trees were neither significantly worse in the MP-based Kishino–Hasegawa test as implemented in PAUP^*^ (α = 0.01) nor in the ML-based Shimodaira–Hasegawa test as implemented in RAxML (α = 0.01), indicating no conflict between the genomic data and 16S rRNA gene. One of the best constrained MP trees is shown in Figure 3 together with ML and MP bootstrap support values from both constrained and unconstrained analyses. Without the constraint, the 16S rRNA gene yielded little overall support, but it maximally supported the sister-group relationship of *P. leonis* with *P. arcticus*. *L. nanhaiensis* was placed apart from the other *Leisingera* species, but without support, not even in the constrained analysis. Importantly, none of the species not present in the phylogenomic analysis were additionally placed in the clade containing the other *Leisingera* species together with *P. caeruleus* and *P. daeponensis*, even though this was not enforced by the constraint.

Again, the topology (Figure 3) differed to some degree from those inferred from the other data matrices, but it also showed a weakly (72/65%) supported monophyletic group containing *P. daeponensis, P. caeruleus, P. aquaemixtae, L. methylohalidivorans*, and *L. aquimarina* as well as another clade containing *P. gallaeciensis* and *P. inhibens* (93/99% support). Further, as in the previous trees, *L. nanhaiensis* was shown to branch first, before *Ruegeria* spp. Thus, none of the branches in conflict with the previous trees obtained particularly high bootstrap supported. The 16S rRNA gene sequences also clearly separated *L. nanhaiensis* from the other *Leisingera* species but yielded few support otherwise.

**Figure 4:**
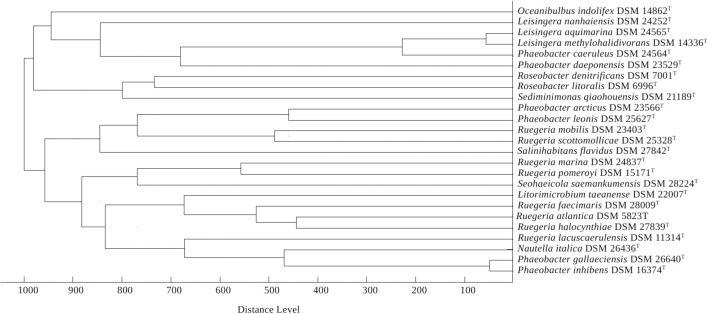
**Score-oriented dendrogram generated by the BioTyper software (version 3.1, Bruker Daltonics) showing the similarity of MALDI-TOF mass spectra of cell extracts of type strains of selected species within the genera *Leisingera, Phaeobacter, Litorimicrobium, Nautella, Oceanibulbus, Roseobacter, Salinihabitans, Seohaeicola, Sediminimonas*, and *Ruegeria***.

The dendrogram from the MALDI-TOF MS analysis is shown in Figure 4. It confirms the following groupings of taxonomic interest in the current study: *P. arcticus* as sister group of *P. leonis*, both set apart from the type species *P. gallaeciensis* and other *Phaeobacter* species; *P. gallaeciensis* as sister group of *P. inhibens*; a group comprising *P. caeruleus, P. daeponensis, L. aquimarina* and the type species *L. methylohalidivorans*; and *L. nanhaiensis* set apart from the other *Leisingera* species and also from *L. taeanense.* Rather, *L. nanhaiensis* is found as sister taxon of the group *P. inhibens* and *P. gallaeciensis.*

An overview of the taxonomically relevant phenotypic characters collected from the literature and the G+C content values inferred from the genome sequences is given in Table 1. They are discussed in detail below in the light of the phylogenetic analyses. Supplementary File 2 contains the complete set of phenotypic characters analyzed in the course of this study.

**Table 1:** **Major phenotypic and genomic properties that differentiate *Leisingera* spp., *Phaeobacter* spp., *Pseudophaeobacter* spp., and *Sedimentitalea nanhaiensis* from each other and from probably closely related genera**.

**Character**	**1 (Ph. arct.)**	**2 (Ph. leon.)**	**3 (Ph. gall.)**	**4 (Ph. inhi.)**	**5 (Le. aqui.)**	**6 (Le. meth.)**	**7 (Ph. aqua.)**	**8 (Ph. caer.)**	**9 (Ph. daep.)**	**10 (Le. nanh.)**	**11 (Li. taea.)**	**12 (Se. saem.)**	**13 (Pe. lito.)**	**14 (Pu. anta.)**
Motility	+	+	+	+	+	+	–	+	+	+	–	–	–	–
Colony color	Yellow	Beige	Brown	Dark-brown	Dark-beige-pink	None	Yellowish-white	Blue	Yellowish-white	Beige	White	Pale-yellow	N.D.	Pink- to -red
G+C content	59.27	58.8	59.44	60.02	61.35	62.33	64.6	63.27	64.34	60.74	62.4	63.4	47.0	60.7
Sole nitrogen source: Nitrate	–	–	–	–	–	–	N.D.	+	+	–	+	+	–	N.D.
**SUSCEPTIBILITY TO ANTIBIOTICS**														
Ampicillin	N.D.	+	N.D.	N.D.	N.D.	N.D.	+	N.D.	+	+	–	+	N.D.	+
Carbenicillin	N.D.	N.D.	N.D.	N.D.	N.D.	N.D.	+	N.D.	+	+	–	+	N.D.	N.D.
Cephalothin	N.D.	N.D.	N.D.	N.D.	N.D.	N.D.	+	N.D.	+	–	N.D.	+	N.D.	N.D.
Gentamicin	N.D.	N.D.	+	±	–	±	+	+	+	–	–	+	N.D.	+
Kanamycin	N.D.	+	+	+	N.D.	±	+	N.D.	+	+	–	+	N.D.	+
Penicillin G	N.D.	+	±	+	N.D.	+	+	N.D.	+	–	+	+	N.D.	+
Polymyxin B	N.D.	N.D.	N.D.	N.D.	N.D.	N.D.	–	N.D.	+	+	–	–	N.D.	N.D.
Streptomycin	N.D.	+	+	±	+	+	+	+	+	–	+	+	N.D.	N.D.
**PHOSPHOLIPIDS**														
DPG	–	+	–	–	N.D.	–	±	N.D.	–	–	+	–	–	–
PC	+	+	+	+	N.D.	–	+	N.D.	+	–	+	+	+	+
PE	+	+	+	+	N.D.	+	+	N.D.	+	+	–	+	+	+
**FATTY ACIDS**														
C10:0 3-OH	3.2–6.8	1.8	1.9–3.2	1.7–2.4	2.0	1.8–6.5	1.6	1.7–3.2	1.7–2.5	3.9–9.4	0.0	0.0	4.0	0.0
C12:0 3-OH	0.0–0.1	0.0	1.3–2.5	1.6–2.0	2.1	2.1–6.4	3.0	2.2–2.9	2.4–2.9	2.9–4.5	0.0	0.0	0.3	0.5
C12:1 3-OH	0.0	0.0	0.0	0.0	0.0	0.0	0.0	0.0	0.0	0.0	9.8	0.0	0.0	4.0

G+C content values were calculated from the genome sequence where possible. Strains: 1, Phaeobacter arcticus DSM 23566^T^ (Zhang et al., 2008; Vandecandelaere et al., 2009; Freese et al., 2013; Liu et al., 2014); 2, Phaeobacter leonis 306^T^ (Gaboyer et al., 2013); 3, Phaeobacter gallaeciensis CIP 105210^T^ (Ruiz-Ponte et al., 1998; Martens et al., 2006; Yoon et al., 2007; Vandecandelaere et al., 2008, 2009; Lucena et al., 2012; Frank et al., 2014; Liu et al., 2014); 4, Phaeobacter (Ruiz-Ponte et al., 1998; Martens et al., 2006; Yoon et al., 2007; Vandecandelaere et al., 2008, 2009; Lucena et al., 2012; Frank et al., 2014; Liu et al., 2014); 4, Phaeobacter inhibens T5^T^ (Martens et al., 2006; Yoon et al., 2007; Vandecandelaere et al., 2008, 2009; Lucena et al., 2012; Dogs et al., 2013b); 5, Leisingera aquimarina DSM 24565^T^ (Vandecandelaere et al., 2008, 2009; Sun et al., 2010; Riedel et al., 2013); 6, Leisingera methylohalidivorans MB2^T^ (Schaefer et al., 2002; Martens et al., 2006; Yoon et al., 2007; Vandecandelaere et al., 2008; Sun et al., 2010; Jin et al., 2011; Buddruhs et al., 2013a); 7, Phaeobacter aquamixtae SSK6-1^T^ (Park et al., 2014); 8, Phaeobacter caeruleus 13^T^ (Vandecandelaere et al., 2009; Beyersmann et al., 2013; Park et al., 2014); 9, Phaeobacter daeponensis TF-218^T^ (Yoon et al., 2007; Vandecandelaere et al., 2008, 2009; Lucena et al., 2012; Dogs et al., 2013a; Park et al., 2014); 10, Leisingera nanhaiensis NH52F^T^ (Sun et al., 2010; Jin et al., 2011; Breider et al., 2014); 11, Litorimicrobium taeanense G4^T^ (Jin et al., 2011); 12, Seohaeicola saemankumensis SD-15^T^ (Yoon et al., 2009); 13, Pelagicola litoralis CL-ES2^T^ (Kim et al., 2008); 14, Puniceibacterium antarcticum SM1211^T^ (Liu et al., 2014). Abbreviations used: DPG, Diphosphatidylglycerol; PC, Phosphatidylcholine; PE, Phosphatidylethanolamine; N.D., Not Determined.

## Discussion

The conducted phylogenomic analyses aimed at generating several distinct genome-scale data matrices for assessing whether, and in which respects, their distinct analyses corroborated each other, instead of generating a single data matrix and subsequent phylogenetic inference whose sensitivity to issues such as gene selection remained essentially unknown (Klenk and Göker, 2010). The conducted analyses indeed corroborated each other regarding the genealogy of the organisms investigated, as no well-supported conflict between the resulting topologies was found. The sole exception might be the single branch in Figure 1 that was not maximally supported by all analyses, but for taxonomic classifications inferred from phylogenomic analyses a remaining set of only ambiguously supported branches would not matter anyway, as not all subtrees could be assigned a taxon name in a Linnean system because of its limited number of taxonomic ranks (Wiley and Lieberman, 2011).

Importantly, the paired-site tests indicate that there is no significant conflict of the topologies from phylogenomic analyses with the 16S rRNA gene data either. The conflicts between the current classification are thus due to the insufficient resolution of the 16S rRNA gene in this group (Figure 3). This problem can hardly be avoided in current microbial taxonomy as long as it relies only on 16S rRNA gene data for phylogenies, simply because newly described species must be placed in some genus. Whereas one might be tempted to presume that stability in taxonomic classification might come from using a standardized set of characters (such as the 16S rRNA gene, or any other standardized set of genes), this is not actually the case. As in previous studies (Spring et al., 2010; Anderson et al., 2011; Göker et al., 2011; Abt et al., 2012, 2013; Frank et al., 2014; Stackebrandt et al., 2014; Verbarg et al., 2014) and listed in Supplementary File 3, the analyses conducted here confirmed that more characters (up to entire genomes) yield better resolved phylogenies (Figure 1) and thus better substantiated classifications. This is particularly striking for the two 31-gene data sets (Ciccarelli et al., 2006; Wu and Eisen, 2008) which, even though the gene sets are partially overlapping, yield distinct phylogenies (Figure 1). These observations strongly argue for a “total evidence” approach (Kluge, 1989; Lienau and DeSalle, 2009). Of course the trees should also be correct, but it is possible to detect artifacts such as long-branch attraction (Bergsten, 2006), and there is no indication for those in the current analyses. In the following we discuss the phylogenomic outcomes in the light of the phenotypic data known for *Leisingera, Phaeobacter* and their probable close relatives and assess several possible taxonomic rearrangements.

In all phylogenetic analyses conducted, *L. nanhaiensis* was unambiguously distinct from the remaining species of the genus, which had a closer relationship to both *Phaeobacter* and *Ruegeria*. In contrast to the other *Leisingera* species, *L. nanhaiensis* shows a lower DNA G+C content and no susceptibility to the antibiotic streptomycin (Table 1). In the original 16S rRNA gene analysis conducted by Sun et al. (2010), *L. nanhaiensis* was placed on a long branch as sister group of the other *Leisingera* species, but with low bootstrap support. The inference method used was neighbor joining based on a Kimura-2-parameter evolutionary model, which might be too simplistic for these data (for instance, it does not distinguish between rapidly and slowly evolving alignment positions) (Felsenstein, 2004). Another reason for the differences between the outcomes of the 16S rRNA gene analyses might be distinct taxon sampling. The 16S rRNA gene tree topologies inferred in the studies on the *Leisingera* and *Phaeobacter* genomes varied considerably depending on the included species from other genera (Beyersmann et al., 2013; Buddruhs et al., 2013a; Dogs et al., 2013a,b; Freese et al., 2013; Riedel et al., 2013; Breider et al., 2014). This is as expected, since the 16S rRNA gene trees for the group hardly contain branches with relevant support. Quite in contrast, the phylogenomic analyses yielded high support, and there is no reason to assume that any of these branches could be easily affected by taxon sampling. As mentioned above, more characters yield better resolved phylogenies and thus more reliable taxonomic classifications; the present study is no exception from this rule. Thus, there is no evidence for the sister-group relationship of *L. nanhaiensis* to the type species *L. methylohalidivorans* and the other *Leisingera* species and evidence from more than a million characters against it (Figure 1).

A potential solution is to include *L. nanhaiensis* in another already established genus of *Rhodobacteraceae*. But given the already evident taxonomic problems in the *Roseobacter* group that have been caused by a low resolution of the 16S rRNA gene, the mere fact that no sister-group relationship between *L. nanhaiensis* and another genus has any statistical support argues against this proposal. Including *L. nanhaiensis* in an already existing genus would avoid introducing a novel genus, but would be too risky, because future phylogenomic studies might easily demonstrate such a group to be non-monophyletic. Characters other than the 16S rRNA gene neither support such a merging. For instance, the MALDI-TOF MS analysis is in conflict with a sister-group-relationship of *L. nanhaiensis* and *L. taeanense* (Figure 4); the analysis would rather suggest an affiliation to *P. gallaeciensis* and *P. inhibens*, but this would be in strong conflict with the phylogenomic analyses (Figures 1, 2). Chemotaxonomically, *L. nanhaiensis* can be distinguished from *L. taeanense* G4^T^ by the presence of phosphatidylethanolamine and C10:0 3-OH (about 4–9%), C12:0 3-OH (about 3–5%) in only the former and the presence of (about 10%) C12:1 3-OH, phosphatidylcholine and diphosphatidylglycerol in only the latter (Table 1). Moreover, they can be distinguished by their motility, anaerobic growth with nitrate and the susceptibility to ampicillin, carbenicillin, kanamycin, penicillin G, polymyxin B and streptomycin (Table 1). Similarly, *L. nanhaiensis* can be distinguished from *S. saemankumensis* regarding anaerobic growth with nitrate, motility, fatty acids and polar lipids and susceptibility/resistance to antibiotics (Table 1). The fatty acid composition of *L. nanhaiensis* is quite similar to the one of *P. litoralis* but the two can be distinguished by the >10% higher G+C content and the absence of phosphatidylcholine in the former (Table 1). Differences between *L. nanhaiensis* and *Puniceibacter antarcticum* are the motility of the former, the presence of (about 4%) C12:1 3-OH in the latter, and the colony color (Table 1). Other characteristics which distinguish *L. nanhaiensis* from other species are shown in Table 1. The MALDI-TOF MS analysis shows *L. nanhaiensis* as sister taxon of the group *P. inhibens* and *P. gallaeciensis.* However, no other evidence (16S rRNA, Figure 4; phenotypic characteristics, Table 1) for including *L. nanhaiensis* in *Phaeobacter* was found.

The differences mentioned above might partially be regarded as few, but one should not overlook that given the frequently ambiguous phenotypic characters in the entire *Roseobacter* group, it is unlikely that, on average, more phenotypic differences would appear if *L. nanhaiensis* would be compared to any other genus within the group. This is supported by the suggested “mix-and-match” genome arrangement (Moran et al., 2007) found within the *Roseobacter* group, which could make the genomes of organisms of this group very flexible. Accordingly the physiology of the organisms is not necessarily reflected by their phylogeny, and it has been found that trophic strategies correlate better than phylogeny (Newton et al., 2010).

The third potential alternative for creating monophyletic taxa, i.e., to merge even more genera (such as all ingroup genera in Figure 3), can be rejected for two reasons. First, it would be less taxonomically conservative as it involved more name changes. Second, given the large number of unsupported branches in the 16S rRNA analysis (Figure 3), merging all these genera would be phylogenetically even more uncertain than the inclusion of *L. nanhaiensis* into another genus. We thus propose to reclassify *L. nanhaiensis* as *Sedimentitalea nanhaiensis* gen. nov., comb. nov. The description of the genus *Leisingera* is emended accordingly.

The discrepancies between phylogeny and classification that remain after the removal of *L. nanhaiensis* from the genus *Leisingera* (Figures 1–3) could be solved either by merging *Leisingera* and *Phaeobacter* (solution I) or by assigning *P. arcticus* and *P. leonis* to a new genus and reclassifying *P. aquaemixtae, P. caeruleus*, and *P. daeponensis* as members of the genus *Leisingera*. As *Leisingera* has priority over *Phaeobacter*, solution I would involve one change at the genus level (removal of the genus *Phaeobacter*) and seven changes at the species level (assignment of the seven *Phaeobacter* species to *Leisingera*). In contrast, solution II would involve one change at the genus level (introduction of one new genus) and five changes at the species level (assignment of five *Phaeobacter* species to another genus). Solution II thus involves fewer overall taxonomic changes. Another disadvantage of solution I is that the clade comprising only *Phaeobacter* and *Leisingera* except for *L. nanhaiensis* is unsupported in the 16S rRNA gene analysis (Figure 3). That is, a merging of *Leisingera* (except *L. nanhaiensis*) and *Phaeobacter* would bear the risk of creating a group that turns out as non-monophyletic once more genomes from the other organisms now included only in the 16S rRNA tree (Figure 3) become available for a phylogenomic analysis. The MALDI-TOF MS analysis (Figure 4) is also in conflict with the merging of the two genera. Furthermore, solution I would lead to a combination of organisms with very different physiological and chemotaxonomic features within one genus. The number of common features for the different species would be reduced and the genus description could only comprise very general features, also found for species of other genera within the *Rhodobacteracae* and thus not suitable for a discrimination. Indeed, as shown in the following, the genus *Phaeobacter* as currently circumscribed is already phenotypically very heterogeneous.

*P. gallaeciensis* and *P. inhibens* produce a brown, diffusible pigment and show a strong inhibitory activity against bacteria, based on production of the antibiotic tropodithietic acid (TDA) (Martens et al., 2006). This is in contrast to what was described for *P. aquaemixtae, P. caeruleus* and *P. daeponensis* (see Yoon et al., 2007; Vandecandelaere et al., 2009; Park et al., 2014 and Table 1). The colony color of these species was described as yellowish-white for *P. aquaemixtae* and *P. daeponensis* but blue for *P. caeruleus*, although *P. daeponensis* also forms blue colonies when grown on YTSS medium (Dogs et al., 2013a). *P. arcticus* and *P. leonis* show a yellow or beige colony color in contrast to the brown or dark brown color described for *P. gallaeciensis* and *P. inhibens* (Table 1). A yellow-brown extracellular pigment is correlated with the production of TDA in members of the *Roseobacter* group (Geng et al., 2008; Berger et al., 2011). Thus, it can be assumed that *P. aquaemixtae, P. arcticus, P. caeruleus, P. daeponensis*, and *P. leonis* do not produce TDA, which is confirmed by the absence of genes involved in TDA production in the known genomes of these organisms. Other phenotypical differences which distinguish *P. gallaeciensis* and *P. inhibens* on the one hand from *P. aquaemixtae, P. caeruleus, P. daeponensis, L. aquimarina*, and *L. methylohalidivorans* on the other hand are the utilization of D-mannose, D-maltose, D-cellobiose, D-galactose, D-xylose and the tolerance to ampicillin (Supplementary File 2). The incapability to utilize D-mannose and D-maltose was previously included in the description of the genus *Leisingera* (Vandecandelaere et al., 2009). It is more difficult to separate *P. arcticus* and *P. leonis* from *Leisingera* phenotypically, but the two are the sole species in the genera *Phaeobacter* and *Leisingera* which hardly or not at all form the fatty acid C12:0 3-OH. Moreover, *P. arcticus* and *P. leonis* are phylogenetically (Figures 1–3), regarding the MALDI-TOF MS data (Figure 4) and the G+C content (Table 1) obviously distinct from *Leisingera*.

*P. aquaemixtae, P. caeruleus*, and *P. daeponensis* also show a higher G+C content (63–64%) than *P. gallaeciensis* (59.4%) and *P. inhibens* (60%). Besides the phylogenetic analyses, showing that *P. aquaemixtae, P. caeruleus*, and *P. daeponensis* cluster together with *L. methylohalidivorans* (the type species of *Leisingera*) and *L. aquimarina*, the phenotypic and genomic characteristics of these three *Phaeobacter* species are also more similar to those of *L. aquimarina* with its dark beige-pink color and G+C content of 61.3%, and of *L. methylohalidivorans*, which is non-pigmented and has a G+C content of 62.3% (Table 1). *P. gallaeciensis* CIP 105210^T^ was tested negatively for genes coding for *tmm* or gammaglutamylmethylamide synthetase (*gmaS*). These genes were used as functional markers for the utilization of methylated amines as alternative nitrogen source (Chen, 2012). *L. aquimarina* and *L. methylohalidivorans* were both tested positively for the two genes and are able to use monomethylamine (MMA) and trimethylamine (TMA) as sole nitrogen source (Chen, 2012). The genomes of *P. caeruleus* and *P. daeponensis* also possess *tmm* and *gmaS* sequences, in contrast to the strains of *P. inhibens* (i.e., T5^T^, 2.10, DSM 17395) and *P. gallaeciensis* (CIP105210^T^) (Chen, 2012; this study).

The conventional DDH experiments conducted by Vandecandelaere et al. (2009) resulted in the highest similarity of *P. caeruleus* with *L. methylohalidivorans* (55 ± 1%). A significantly lower similarity (40 ± 5%) was found with *P. gallaeciensis*, indicating a closer affiliation to the genus *Leisingera*. A comparison with DDH similarities calculated *in silico* for genome-sequenced strains using GGDC 2.0 supports the results of the phylogenomic analyses. Comparatively high similarity (36.2 ± 2.57) was observed between *P. gallaeciensis* CIP 105210^T^ and *P. inhibens* T5^T^, compared to values between 20.6 ± 2.46 and 22.6 ± 2.46 for the similarities to the other species of the genera *Phaeobacter* and *Leisingera* (Dogs et al., 2013b). The similarities between the strains *L. aquimarina* DSM 24565^T^, *L. methylohalidivorans* MB2^T^, *P. caeruleus* 13^T^, and *P. daeponensis* TF-218^T^ ranged from 27.9 ± 2.43 to 40.3 ± 2.51, compared to lower values of 19.2 ± 2.28 to 21.5 ± 2.34 for the similarities to the other strains (Beyersmann et al., 2013; Buddruhs et al., 2013a; Dogs et al., 2013a; Riedel et al., 2013). The DDH result for *L. aquimarina* DSM 24565^T^ and *L. methylohalidivorans* MB2^T^ was 32.4 ± 2.46 (Riedel et al., 2013), the result for *P. caeruleus* 13^T^ and *P. daeponensis* TF-218^T^ was 40.3 ± 2.51 (Beyersmann et al., 2013), in accordance with the branching within the trees (Figures 1–3). The *in silico* DDH analysis of *P. arcticus* DSM 23566^T^ showed only low similarity values (20.4 ± 2.32 to 22.9 ± 2.37) when compared to the other *Phaeobacter* and *Leisingera* strains (Freese et al., 2013), indicating a lower degree of relatedness.

Based on these polyphasic results we propose to reclassify *P. aquaemixtae* as *Leisingera aquaemixtae* comb. nov., *P. caeruleus* as *Leisingera caerulea* comb. nov., *P. daeponensis* as *Leisingera daeponensis* comb. nov., *P. arcticus* as type species of the new genus *Pseudophaeobacter* as *Pseudophaeobacter arcticus* gen. nov., comb. nov., and *P. leonis* as *Pseudophaeobacter leonis* comb. nov. The descriptions of the genera *Phaeobacter* and *Leisingera* are emended accordingly.

The proposed reclassifications lead to a homogenous *Leisingera*-*Phaeobacter* cluster, consisting of monophyletic genera for *Phaeobacter* (including only *P. gallaeciensis* and *P. inhibens*) and *Leisingera* (including *L. aquaemixtae, L. aquimarina, L. caerulea, L. daeponensis* and *L. methylohalidivorans*) with *Pseudophaeobacter* (including *P. arcticus* and *P. leonis*) and *Sedimentitalea* (including *S. nanhaiensis*) as separate lineages. Reduction of the genus *Phaeobacter* to the species *P. gallaeciensis* and *P. inhibens* returns to the original genus description given by Martens et al. (2006) and allows a much better discrimination of the genus *Phaeobacter* against the closely related genera. Equivalently, the changes suggested in solution II allow for a better discrimination of the genus *Leisingera* and the newly proposed genera *Pseudophaeobacter* and *Sedimentitalea*. Classification of new species would subsequently be based on much clearer taxonomic definitions. Given the low resolution of the 16S rRNA gene within *Rhodobacteraceae*, more precisely defined genera will also reduce the future risk of creating non-monophyletic groups.

### Description of *sedimentitalea*, gen. nov.

*Sedimentitalea* (Se.di.men.ti.ta'le.a. L. n. *sedimentum*, sediment; L. fem. n. *talea*, a rod; N.L. *Sedimentitalea*, fem. a rod isolated from sediment).

Gram negative, oxidase and catalase positive, motile rod-shaped bacteria, 0.6–0.8 μm wide and 1.6–3.0 μm long. Sodium ions are essential for growth. The major polar lipids are phosphatidylglycerol, phosphatidylethanolamine, an unidentified phospholipid, an unidentified lipid and an aminolipid. The fatty acid composition (>1%) is C18:1 ω 7*c*, an unknown fatty acid (equivalent chain length of 11.799), C16:0 2-OH, C10:0 3-OH, C16:0, 11-methyl C18:1 ω 7*c* and C12:0 3-OH. The G+C content is about 60–61%.

On the basis of 16S rRNA gene sequence and particularly phylogenomic analysis, the genus represents a separate branch within the family *Rhodobacteraceae* of the class *Alphaproteobacteria*. The type species (and currently sole species) of the genus is *S. nanhaiensis*.

### Description of *sedimentitalea nanhaiensis*, comb. nov.

*S. nanhaiensis* (nan.hai.en'sis. N.L. fem. adj. *nanhaiensis* referring to Nanhai, the Chinese name for the South China Sea, from where the type strain was isolated).

Basonym: *Leisingera nanhaiensis* (Sun et al., 2010).

The description is the same as for *L. nanhaiensis* (Sun et al., 2010) as emended by Breider et al. (2014). The type strain is NH52F^T^ (LMG 24841^T^, DSM 24252^T^).

### Description of *pseudophaeobacter*, gen. nov.

*Pseudophaeobacter* (Pseu.do.phae.o.bac'ter. Gr. adj. *pseudes* false; N.L. masc. n. *Phaeobacter*, a bacterial genus; N.L. masc. n. *Pseudophaeobacter* false *Phaeobacter*).

Gram-negative, aerobic, oxidase and catalase positive, rod-shaped bacteria. The cells are 1.0–2.6 μm long and 0.3–0.5 μm wide. Sodium ions are essential for growth. Ubiquinone-10 is the principal isoprenoid quinone. The main polar lipids present are phosphatidylethanolamine, phosphatidylglycerol, phosphatidylcholine and an unidentified aminolipid. The predominant fatty acids (>1%) are C18:1 cis7ω, C18:1 cis7ω methyl, an unknown fatty acid (equivalent chain length of 11.799), C16:0 (hexadecanoic acid), C10:0 3-OH. The G+C content of the currently two species of this genus is 58.8–59.2%.

On the basis of 16S rRNA gene sequence and particularly phylogenomic analysis, the genus represents a separate branch within the family *Rhodobacteraceae* of the class *Alphaproteobacteria*. The type species is *P. arcticus*.

### Description of *pseudophaeobacter arcticus*, comb. nov.

*P. arcticus* (arc'ti.cus. L. masc. adj. *arcticus* northern, arctic, referring to the site from where the type strain was isolated).

Basonym: *Phaeobacter arcticus* (Zhang et al., 2008).

The description of the species is the same as given for *P. arcticus* by Zhang et al. (2008). The type strain is 20188^T^ (JCM 14644^T^, DSM 23566^T^).

### Description of *pseudophaeobacter leonis*, comb. nov.

(le.o'nis., L. gen n. *leonis*, of a lion, named after sinus Leonis, the Medieval Latin name of the Gulf of Lion, in reference to the origin of the type strain).

Basonym: *Phaeobacter leonis* (Gaboyer et al., 2013).

The description of the species is the same as given for *P. leonis* by Gaboyer et al. (2013). The type strain is 306^T^ (DSM 25627^T^, CIP 110369^T^).

### Emended description of the genus *leisingera* (schaefer et al., 2002)

*Leisingera* (Lei.sin'ge.ra. N.L. fem. n. *Leisingera* in honor of Thomas Leisinger, on the occasion of his retirement and for his contributions to our understanding of the biochemistry of bacterial methyl halide metabolism).

The description given by Schaefer et al. (2002) and emended by Martens et al. (2006) and Vandecandelaere et al. (2008) is no longer appropriate due to the addition of further species previously classified in *Phaeobacter.* The description is thus as given by Vandecandelaere et al. (2008) with the following modifications.

Colony color can vary from non-pigmented to yellowish-white to dark beige-pink and blue, depending on the medium used. The G+C content ranges from 61.3 to 64.6%. Does not degrade casein or hydrolyse aesculin. Positive for leucine aramylase activity, but no activity is detected for lipase (C14), cystine arylamidase, trypsin, α-chymotrypsin, α-galactosidase, β-glucoronidase, α-glucosidase, N-acetyl-β-glucosaminidase, α-mannosidase, and α-fucosidase. Do not assimilate D-maltose or D-mannose. Susceptible to streptomycin. Able to utilize methylated amines as alternative nitrogen source. The type species is *L. methylohalidivorans*. Its type strain is MB2^T^ (ATCC BAA-92^T^, DSM 14336^T^).

### Description of *leisingera aquaemixtae*, comb. nov.

*L. aquaemixtae* (a.quae.mi'xtae. L. fem. n. *aqua* water; L. fem. part. adj. *mixta* mixed; N.L. fem. gen. n. *aquaemixtae* of mixed waters).

Basonym: *Phaeobacter aquaemixtae* (Park et al., 2014).

The description is the same as that given for *P. aquaemixtae* (Park et al., 2014). The type strain is SSK6-1^T^ (KTCC 32538^T^, CECT 8399^T^).

### Description of *leisingera caerulea*, comb. nov.

*L. caerulea* (cae.ru'le.a. L. fem. adj. *caerulea* dark blue colored, referring to the colony color of the isolates).

Basonym: *Phaeobacter caeruleus* (Vandecandelaere et al., 2009).

The description is the same as that for *P. caeruleus* (Vandecandelaere et al., 2009). The type strain is LMG 24369^T^ (CCUG 55859^T^, DSM 24564^T^).

### Description of *leisingera daeponensis*, comb. nov.

*L. daeponensis* (dae.po.nen'sis. N.L. fem. adj. *daeponensis* of Daepo, Korea, where the type strain was isolated).

Basonym: *Phaeobacter daeponensis* (Yoon et al., 2007).

The description is the same as that for *P. daeponensis* (Yoon et al., 2007) as emended by Vandecandelaere et al. (2008) and Dogs et al. (2013a). The type strain is TF-218^T^ (KCTC 12794^T^, DSM 23529^T^).

### Emended description of the genus *phaeobacter* (martens et al., 2006)

The emended description given by Yoon et al. (2007) is no longer appropriate due to the reclassification of *P. daeponensis* as *L. daeponensis*. The description is thus as given by Martens et al. (2006) with the following modifications.

*Phaeobacter* colonies are brownish to dark brown and a diffusible brownish pigment is produced. Produce TDA. Nitrate is not reduced. Facultatively anaerobic by reduction of nitrite. The G+C content is in the range 59.4–60.0%. The type species is *P. gallaeciensis*. Its type strain is BS107^T^ = CIP 105210^T^ = DSM 26640^T^.

### Conflict of interest statement

The authors declare that the research was conducted in the absence of any commercial or financial relationships that could be construed as a potential conflict of interest.
