# Supplementary material for: Genome-scale data suggest reclassifications in the Leisingera-Phaeobacter cluster including proposals for Sedimentitalea gen. nov. and Pseudophaeobacter gen. nov
Source: Front Microbiol. 2014 Aug 11;5:416. doi: 10.3389/fmicb.2014.00416 (PMC4127530; doi:10.3389/fmicb.2014.00416)
Supplement: Supplementary file 1 [file DataSheet1.ZIP › Supp Mat legends.pdf]

Supplementary Material

Supplementary File 1. *Rhodobacteraceae* phylogeny inferred from the 16S rRNA gene matrix under the maximum likelihood (ML) criterion. Rooting was done with *Labrenzia*, *Pannonibacter*, *Pseudovibrio*, *Roseibium* and *Stappia*. The branches are scaled in terms of the expected number of substitutions per site. Numbers above the branches (from left to right) are bootstrapping support values (if larger than 60%) from (i) ML and (ii) maximum-parsimony (MP) analysis.

Supplementary File 2. Spreadsheet containing the complete set of phenotypic characters of organisms analysed in this study.

Supplementary File 3. The method parameters and the resulting data matrix sizes and average bootstrap support values from previous studies (and this study) using the DSMZ phylogenomics pipeline (Abt *et al*., 2012, 2013; Anderson *et al*., 2011; Frank *et al.,* 2014; Göker *et al*., 2011; Spring *et al*., 2010; Stackebrandt *et al.,* 2014; Verbarg *et al.,* 2014).
