# Supplementary figures and images for: Genome-scale data suggest reclassifications in the Leisingera-Phaeobacter cluster including proposals for Sedimentitalea gen. nov. and Pseudophaeobacter gen. nov
Source: Front Microbiol. 2014 Aug 11;5:416. doi: 10.3389/fmicb.2014.00416 (PMC4127530; doi:10.3389/fmicb.2014.00416)

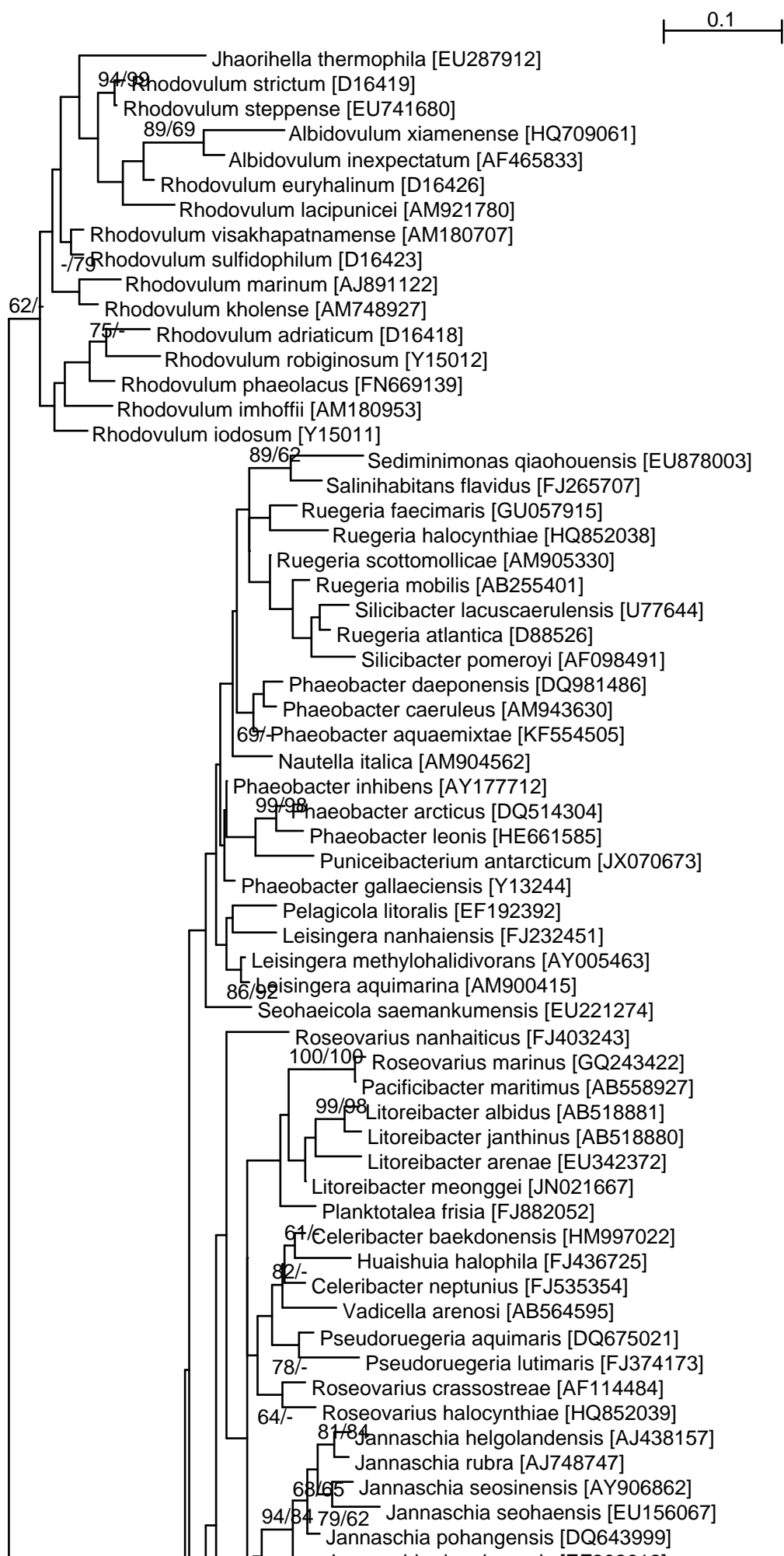

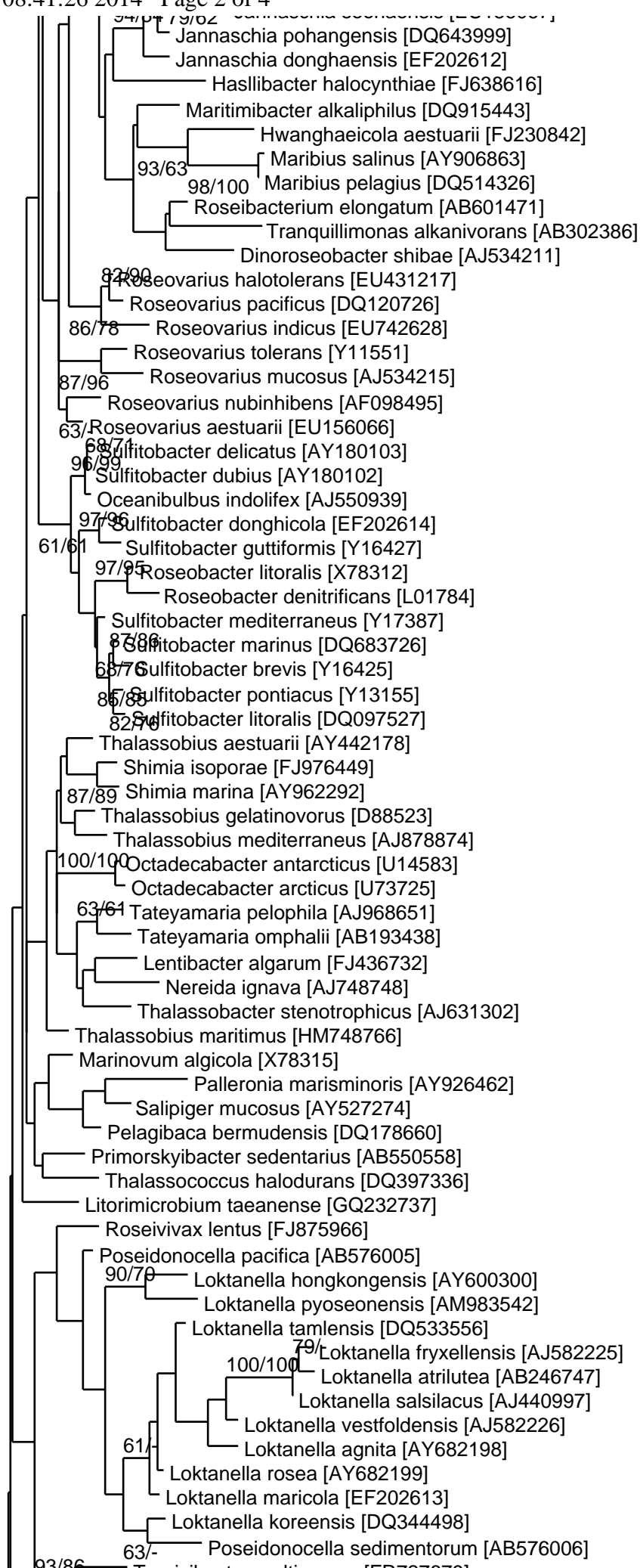

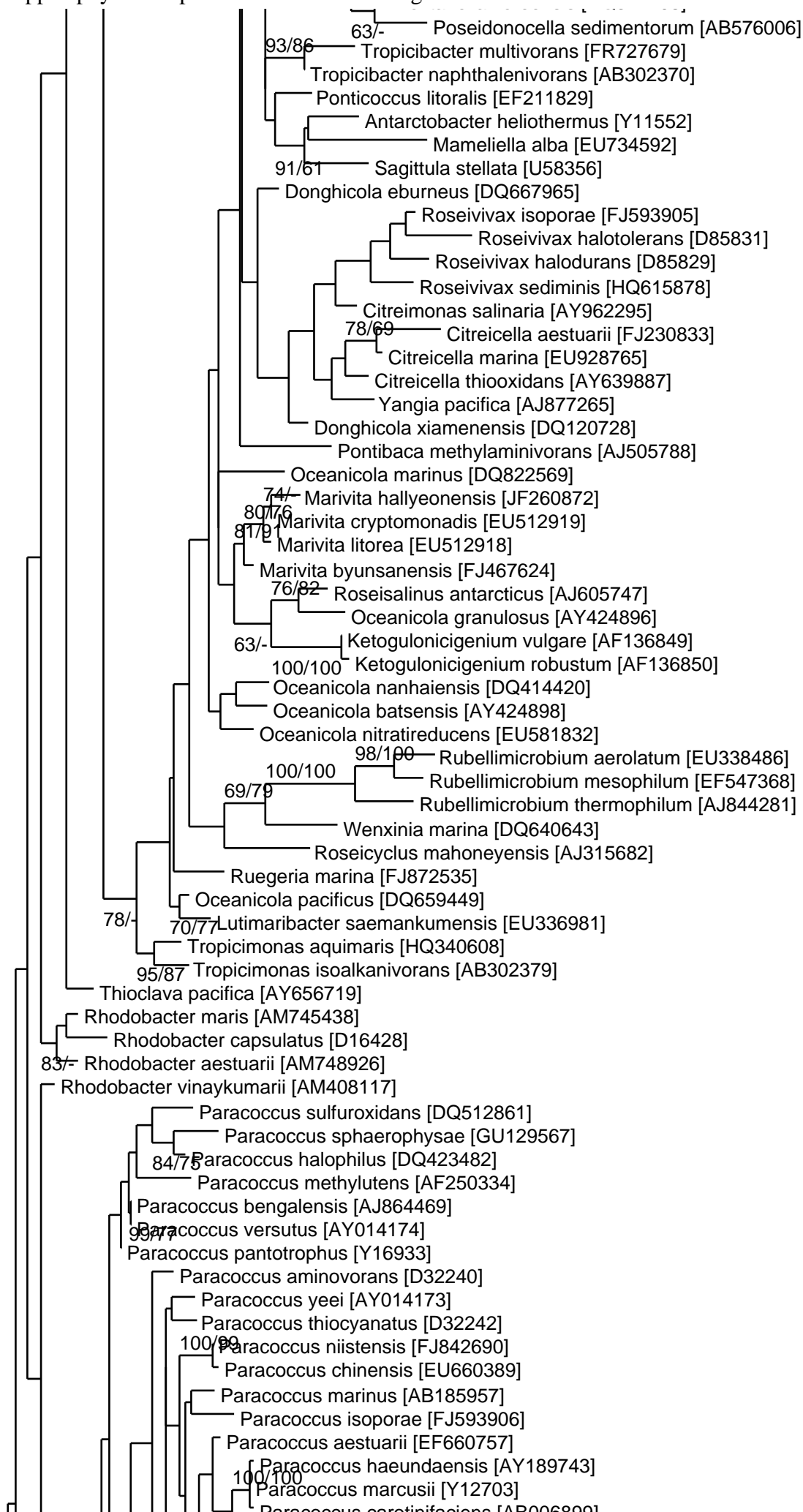

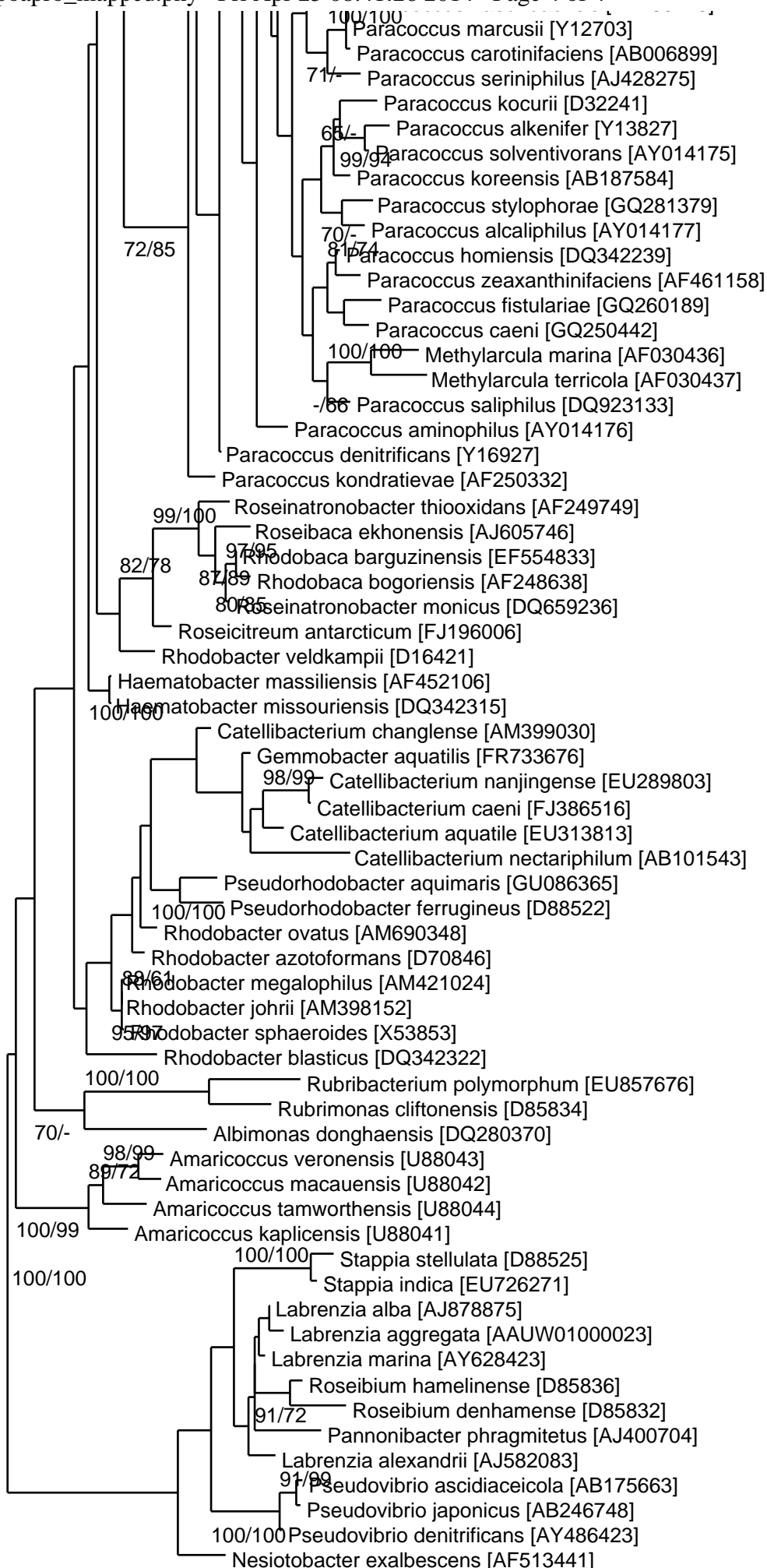

Supplement: Supplementary file 1 [file DataSheet1.ZIP › Supp Mat Datasheet 1.PDF]
